# Crowdsourcing to develop open-access learning resources on antimicrobial resistance

**DOI:** 10.1186/s12879-021-06628-0

**Published:** 2021-09-06

**Authors:** Eneyi E. Kpokiri, Randall John, Dan Wu, Noah Fongwen, Jehan Z. Budak, Christina C. Chang, Jason J. Ong, Joseph D. Tucker

**Affiliations:** 1 Faculty of Infectious and Tropical Diseases, London School of Hygiene and Tropical Medicine, Keppel St., Bloomsbury, London, WC1E 7HT UK; 2 Department of Health Policy and Management, Gillings School of Global Public Health, University of North Carolina, Chapel Hill, NC USA; 3 Department of Medicine, Division of Allergy & Infectious Diseases, University of Washington, Seattle, WA USA; 4 Partners ID Images, Department of Infectious Diseases, Massachusetts General Hospital, Boston, MA USA; 5 Central Clinical School, Monash University, Melbourne, Australia; 6 Social Entrepreneurship To Spur Health (SESH), Guangzhou, China; 7 Institute of Global Health and Infectious Diseases, University of North Carolina, Chapel Hill, NC USA

**Keywords:** Antimicrobial resistance, Infectious diseases, Antimicrobial stewardship, Medical education, Curriculum development, Crowdsourcing

## Abstract

**Objectives:**

Antimicrobial resistance (AMR) is a significant threat to global public health. Many medical curricula have limited clinical cases and materials focused on AMR, yet enhanced AMR education and training are needed to support antimicrobial stewardship programmes. We used crowdsourcing methods to develop open-access, learner-centred AMR resources. Crowdsourcing is the process of having a large group, including experts and non-experts, solve a problem and then share solutions with the public.

**Methods:**

We organised a global crowdsourcing contest soliciting AMR-related multiple-choice questions, infographics, and images. First, we convened a diverse steering committee group to finalise a call for entries. Second, we launched the contest and disseminated the call for entries using social media, blog posts, email, and an in-person event. Partner institutions included two digital healthcare platforms: Figure 1^®^ and Ding Xiang Yuan. Both organizations serve as online communities for healthcare specialists and professionals to report and comment on clinical information. At the end of the call, solicited entries were screened for eligibility and judged on merit and relevance to AMR learning and education. Exceptional entries were recognised, awarded prizes, and further reviewed for sharing with the public via open-access platforms.

**Results:**

We received 59 entries from nine countries. These included 54 multiple-choice questions, four infographics, and one image. Eligible entries (n = 56) were reviewed and assigned a score on a 1–10 scale. Eight entries received mean scores greater than 6.0 and were selected as finalists. The eight finalist entries consisted of three infographics and five multiple-choice questions. They were disseminated through open-access publications and online medical communities. Although we launched a global call, we relied heavily on medical student groups and the entries received were not entirely globally representative.

**Conclusions:**

We demonstrate that crowdsourcing challenge contests can be used to identify infectious disease teaching materials. Medical educators and curriculum developers can adapt this method to solicit additional teaching content for medical students.

**Supplementary Information:**

The online version contains supplementary material available at 10.1186/s12879-021-06628-0.

## Background

Antimicrobial resistance (AMR) is a major global health problem. Antimicrobial stewardship programmes are increasingly designed to enhance and expand medical school infectious disease curricula [1–3]. Educational interventions have been shown to improve antimicrobial use and practices [4, 5]. Educational antimicrobial stewardship can be beneficial to clinicians in high and low-income settings to increase their understanding of AMR. Studies from the United States and Europe suggest gaps in medical student exposure to appropriate antimicrobial prescribing practices and AMR [6, 7]. In addition, separate surveys in the Congo and Ethiopia suggest poor levels of AMR understanding among healthcare providers and students [8, 9]. Other studies from the United Kingdom and Belgium also demonstrate that there are inconsistencies between antibiotic prescribing guidelines and clinician practices [9, 10]. To address AMR, the World Health Organization (WHO) suggests implementing more vigorous educational models and training for healthcare providers [11]. In response, we sought to identify medical education resources on AMR using crowdsourcing methods. Crowdsourcing has a large group, including experts and non-experts, solve a problem and then share solutions with the public [12]. In the past, crowdsourcing has been used to expand existing medical curricula and develop flashcard study tools for preclinical education [13–15]. Additionally, researchers have successfully crowdsourced challenging, high quality, and complex multiple-choice questions (MCQs) from medical students [16].

There are several reasons why crowdsourcing is an effective approach to address medical education development and AMR. First, medical curricula development can be an arduous process for medical educators [17]. Crowdsourcing provides a structured mechanism to involve a large number of individuals in the process of curriculum development [17].

Second, crowdsourcing contests allow organizers to engage community members and raise public awareness [12]. Many groups have suggested that AMR public awareness and public engagement are crucial [18–20]. Third, crowdsourcing draws on open science principles that are increasingly important within medical training and research. Fourth, crowdsourcing can engage junior physicians and build out a pipeline of people interested in medical education.

The purpose of this study was to describe a crowdsourcing contest soliciting AMR infographics, MCQs, and images for medical teaching. We solicited MCQ and infographic submissions because 1) MCQs are preferred by some students as study tools, and 2) surveys among healthcare providers suggest a preference for communicating clinical information through infographics in comparison to conventional text reports [16, 21]. Medical educators and curriculum developers can adopt this method in the future to create AMR-focused learning materials for educational and antimicrobial stewardship efforts.

## Methods

The crowdsourcing contest design was based on the framework provided by the UNICEF/UNDP/World Bank/WHO Special Programme for Research and Training in Tropical Diseases (TDR) Practical Guide on Crowdsourcing in Health and Health Research [12]. The WHO framework provides a systematic approach to crowdsourcing within health contexts. Although this framework focuses on the application of crowdsourcing in public health settings more broadly, we specifically sought to assess its use in the area of medical education and training. According to the WHO model, crowdsourcing has six steps: selecting crowdsourcing as the methodology, convening a steering committee, engaging communities to participate, receiving and judging contributions, recognising finalists, and sharing solutions (Table 1).

**Table 1:** Stages employed in the contest

**Organising a steering committee** The purpose of the steering committee was to provide guidance and outline an overall framework for contest execution. We convened a steering committee composed of ten individuals from five countries that met periodically through 60-min teleconference meetings over the duration of the contest to discuss design, organisation, and implementation. Members included experts in medicine, public health, infectious diseases, and medical education, as well as representatives from partner organisations who helped with contest promotion and dissemination	
**Engaging the community to contribute** The contest was officially launched on April 15, 2019. The website provided detailed information on the contest, including the purpose, categories of participation, rules, steering committee members, and partner organisations. In order to foster creativity and avoid cognitive fixation, we did not provide examples of entries. Promotional information was disseminated through social media (Instagram and Twitter), blog posts, email, and personal contacts. Emails were sent to relevant entities and individuals, including medical student interest groups at multiple institutions (such as interest groups in internal medicine, infectious diseases, and global public health), researchers, and medical specialists across multiple countries. We promoted the contest using Figure 1^®^ and Ding Xiang Yuan (DXY). Both are digital platforms that bring together medical students, healthcare providers, and other healthcare-oriented professionals to share, distribute, and comment on medical cases. [18] DXY is China's largest online healthcare community, with more than four million registered users. On DXY’s main platform website, we paid for four banner advertisements and one push notification to registered users, which reached 16,323 individuals and had 99 unique opens. We also created an official Figure 1^®^profile to facilitate contest promotion and developed a promotional infographic that was distributed via the Figure 1^®^ app and made using Canva, an online graphic-design tool In order to encourage participation on Figure 1^®^, we posted two MCQs on their platform. Each question focused on correct antibiotic treatment options based on presenting symptoms. As of November 9, 2019, the first promotional MCQ received 31 user-comments and 13,762 views and the second MCQ received 54 user-comments and 17,429 views. Promotion was also conducted through the official Figure 1^®^ app email account Finally, we promoted the contest through one in-person event. Contest flyers were distributed at an AMR-focused conference in Belfast, United Kingdom where over 150 healthcare professionals working in the field of antimicrobial resistance and infection prevention and control convened We analysed metrics from both Figure 1^®^ and the contest website. Figure 1® metrics showed that there were 85 comments, 107 saves, and 31,191 views from both promotional MCQs. Email analytics from the Figure 1^®^ official email account suggest that details regarding the contest were disseminated to a high number of individuals. This first email had 764 opens, 491 unique opens, and 50 clicks. A digest email that consisted of both AMR contest and Figure 1^®^ app content and was then sent to primary care physicians, nurses, and medical students. This email had 13,485 email opens, 8979 unique clicks, and 74 clicks on AMR contest content. A third email was sent on May 22, 2019 specifically asking Figure 1^®^ users to contribute AMR related MCQs. This email had 19,298 opens, 12,243 unique opens, and 6781 clicks Using online analytical tools, official contest website metrics were obtained between April 30 and June 9, for a total of 41 days. During this period, the website received 578 total clicks, for an average of 14.1 clicks per day, or 98.7 clicks per week. By reviewing IP addresses, it was determined that website clicks originated from 36 different countries	
**Receiving and evaluating contributions** Participants were given the option to submit their entries through a digital form made using Qualtrics^©^ Survey Software (Qualtrics, Provo, UT) or upload their entry through either the Figure 1^®^ app or DXY, provided that they previously had a platform account. Individuals could submit multiple entries. In addition to entries, we collected the following sociodemographic details regarding participants: name, institution, and country. We asked participants to specify a target audience for their entry (general and primary care physicians, medical students, pharmacists, infectious disease specialists, nurses, etc.) and identify any AMR learning objective(s) that were addressed, whether it be in regards to background, prevention, diagnosis, or treatment of AMR (Table **2**). Consent was sought from participants regarding the modification and use of solicited entries for learning purposes, along with records of proper citation of external sources, and documentation of patient confidentiality Eligibility was determined based on pre-specified criteria: that the entry focused on AMR, was in English, and was in the correct format (as either a MCQ accompanied by answers, an infographic accompanied by brief explanatory captions, or an image). After determining eligibility, entries were transferred to evaluators for phase 1 judging. Evaluation was conducted by three clinical experts who were identified by the steering committee. Their participation as a judge was voluntary During phase 1, each judge awarded every entry an individual score between 1 and 10 based on predetermined criteria. Predetermined evaluation criteria included adherence to the required format, contribution to existing learning resources, relevance and effectiveness in enhancing awareness and understanding of AMR, and focus on one or more of four AMR learning objectives (Table **2**). The three individual scores were then averaged to determine one single score. Comments and revisions to further improve and develop finalist entries were sent to participants	
**Recognising finalists** The eight finalists with mean scores of 6.0 or greater were awarded a total of 1000 USD in prize money through Amazon gift cards and cash. Entries were awarded differing cash prize amounts based on the strength of the submission. All submitters were awarded a commendation certificate in recognition of their participation. The judges also received a thank you letter	
**Sharing solutions** After further review by expert judges, entries were shared with the public. The MCQs were arranged into a slide deck and included as an additional study material to an AMR learning module that was developed through a similar challenge contest in 2018 [18] Nine MCQs were selected by Figure 1^®^ to be shared on their platform via posts, reaching a total of 68 comments, 126 saves, and 81,928 views. Three finalist infographics were published as posters on F1000Research, a life sciences-focused digital publishing platform We asked participants to identify a target audience for their entry. Target audiences identified by submitters included medical students, general practitioners and physicians, internal medicine specialists, infectious disease specialists, microbiologists, prescribers in low- and middle-income countries, junior doctors, and nurses. Submitters also identified veterinary practitioners and farmers as a target audience due to the increasing prevalence of antibiotics in agriculture practices and raising livestock	

### The open challenge contest

The International Diagnostics Centre at the London School of Hygiene and Tropical Medicine and SESH (Social Entrepreneurship to Spur Health) organised this contest. The contest was officially launched in April 2019, and the call was open for two months. An open call for entries was provided on a contest website and promoted using the online medical learning platforms and other social media channels. We collaborated with two digital healthcare platforms in order to disseminate contest promotional materials: Figure 1^®^ and Ding Xiang Yuan. Figure 1^®^ is a Toronto-based digital platform that allows health professionals to share and comment on clinical cases [22]. Similarly, Ding Xiang Yuan is a Chinese digital platform that allows physicians to share medical information [23]. We selected these platforms for two reasons. First, the platforms allowed us to promote the contest in multiple languages (English and Chinese) and access potential participants in various geographical locations. Second, both platforms are specifically tailored for and used by clinicians and healthcare providers, which works well for our challenge contest as we sought to engage these particular groups to participate and send entries. After the open call was closed, all submitted entries were first screened for eligibility. Eligible entries were evaluated by three clinical experts who were identified by the steering committee and agreed to serve as contest judges. Each of the three clinical experts assigned entries a single score between 1 and 10 (with 1 denoting the weakest case, and 10 denoting exceptional submissions). The three scores were then averaged to determine a final single score for each entry. Eight entries that achieved a mean final score of 6.0 or greater emerged as finalists and were awarded a total of 1000 USD in gift cards. We selected 6.0 as a predetermined cut-off value to identify finalists as we deemed entries with an average score of ≥ 6.0 to be of relatively high quality and value. After revising entries, the MCQs were arranged into a slide deck similar to an AMR learning module developed through a challenge contest [24]. The finalist infographics were published as posters on F1000Research, a life sciences-focused digital publishing platform [25–27]. We asked participants to specify the AMR learning objective(s) (Table 2) that their entry addressed. Consensus on prioritizing AMR learning objectives were developed through a modified Delphi survey with stakeholders in AMR [28]. The Delphi survey was conducted amongst attendees in a one day AMR symposium that held in London, United Kingdom. These learning objectives also overlap with the Strategic Objectives outlined by the WHO’s Global Action Plan on Antimicrobial Resistance [29]. The contest was organised in line with terms and conditions as specified by the legal committee of the London School of Hygiene and Tropical Medicine (LSHTM). As part of the conditions of the contest, participants were required to obtain informed consent from subjects where any personal data was included in the entry. Ethics approval was deemed unnecessary by the institutional review board at LSHTM.

**Table 2:** AMR learning objectives

Overall objectives	Individual objectives
Background Information	1. Interpret local epidemiologic data or antibiograms to determine local rates of AMR infections 2. List key risk factors for drug-resistant infections
Prevention	3. Describe factors that may lead to unnecessary antibiotic prescribing by healthcare providers 4. Describe the types of precautions needed/infection control measures for AMR organisms
Diagnosis	5. Interpret susceptibility of testing results to select the most appropriate antibiotic regimen 6. Utilise the local (and regional, if available) microbiology lab to help interpret patient test results
Treatment	7. Identify infections that do not require antibiotic therapy 8. Recognise that treatment of infections may require both antibiotic therapy and source control 9. Recognise the concept of using the narrowest spectrum antibiotic for the shortest period of time 10. Utilise a multidisciplinary healthcare approach when managing AMR organism 11. List resources that can be useful in the treatment of patients with AMR infections 12. Describe the incidence and spectrum of adverse antibiotic effects

## Results

We received 59 entries with 56 eligible entries that came from nine countries: Cameroon (n = 30), the United States (n = 10), Nigeria (n = 5), the United Kingdom (n = 3), Australia (n = 3), Jordan (n = 2), Singapore (n = 1), India (n = 1), and China (n = 1). Of the 56 eligible entries, there were 51 MCQs, four infographics, and one image. Of the 56 eligible entries, 54 were solicited through the official contest website, and two were solicited through the online learning platforms.

The average score of all entries (n = 56) was 4.84. Breakdown of final scores by entry type shows that infographics (n = 4) had an average score of 7.00, images (n = 1) had an average score of 5.00, and MCQs (n = 51) had an average score of 4.67.

Finalist entries centred on a wide range of topics in AMR education and research. MCQs selected as finalists focused primarily on AMR background information and prevention/treatment. Topics included effective infection control in health institutions, multi-drug resistant organisms, antibiotic usage in animal farming, antibiotic treatment options in response to persistent symptoms, and mechanisms of AMR. Participants also identified veterinary practitioners and farmers as a target audience due to the increasing prevalence of antibiotics in agriculture practices and raising livestock [23]. The finalist infographics addressed AMR background information, treatment, and diagnosis. Finalist submissions are included in Additional file 1.

## Discussion

We have demonstrated that crowdsourcing methods can be used to identify open-access medical education materials on antimicrobial resistance (AMR). Our findings support existing literature demonstrating that crowdsourcing is a feasible method to develop educational resources in the medical and public health fields. [13, 15, 16] This challenge contest is a unique example of how to implement crowdsourcing methods to create medical education curricula specifically for the purposes of enhancing antimicrobial stewardship efforts.

This study draws on insights and examples from different settings. The contest was global in scope, as we received 56 eligible entries from nine different countries across five continents. We were able to solicit entries from both high-income and low-income countries, as well as entries from different practice areas, such as human medicine, veterinary medicine, and hospital- and community-based medicine. In addition, we were able to identify relevant online platforms to support contest implementation. The use of online platforms facilitated broad dissemination to an international audience and spurred engagement surrounding AMR and antibiotic prescribing practices. The inclusion of several social media metrics in our study also offers key insight into the use of digital platforms in crowdsourcing challenge contests and can guide future contest-organisers who wish to interact with online platforms for contest organisation and promotion.

This challenge contest received high-quality submissions, consistent with other crowdsourcing studies [30]. In our contest, eight entries (representing 14% of all submissions) achieved a mean score of 6.0 or greater, which was similar to the frequency of high-quality submissions in another global health innovation contest on Hepatitis B and C [30]. In terms of entry dissemination, three infographics were identified for online publication through F1000Research, nine MCQs were shared through the online learning platforms and 22 MCQs were selected for inclusion in a study slide deck on AMR. 25 out of 56 eligible entries (representing 43% of the submissions) were selected for dissemination, which was slightly higher than the dissemination frequency from other challenge contests [30, 31].

Crowdsourcing has several advantages. First, through contest promotion, we were able to spur creativity and awareness surrounding AMR and acquired a diverse and global range of ideas. Soliciting MCQs, infographics, and images on AMR from medical students, physicians, and other healthcare professionals in multiple countries suggests that crowdsourcing is also feasible across different settings. Second, medical curricula development can be a time-consuming and challenging task for a small number of individuals [13, 17]. We demonstrate that a bottom-up crowdsourcing approach can be a cost-effective method to develop medical teaching materials rapidly, decreasing the potential burden on educators and curriculum developers. This suggests that similar to other studies, crowdsourced materials from both experts and non-experts can be used in medical education [14, 15, 32]. Consistent with existing literature, our crowdsourcing approach involved coordinating with finalists in order to edit and refine submissions [13]. An important aspect of crowdsourcing is the process of having experts and non-experts work collaboratively in order to arrive at a final solution. Although the process of editing submissions can be more time-consuming, it also has some intrinsic value in terms of spurring engagement and participation across a wide continuum of stakeholders and participants. Future research should investigate methods to further optimize and streamline the process of developing medical education content through crowdsourcing.

Although many people viewed the contest promotion announcements on online platforms, we received few submissions from them: only two entries were submitted through the online platforms. This data suggests that paid online platforms to promote participation in challenge contests may be less effective, indicating a need for additional crowdsourcing interventions studying the use of paid online platforms. Given that in-person promotion of challenge contests has been associated with a greater volume of entries, more attention to in-person activities may also be useful for promotion [33, 34].

Our study has implications for research and policy surrounding medical education and curriculum development. While our study shows how crowdsourcing is an effective strategy to develop additional study resources in medical education, there is a need for more research to evaluate the impact and effectiveness of these educational resources. Robust programmes are essential in evaluating the extent to which the use of medical education stewardship approaches translate into improvements in clinical practice and understanding. There is also a need to review current curricula on AMR to identify content gaps and inform future projects.

Our contest had some limitations. First, we heavily targeted medical student groups. However, the timing of the call for entries overlapped with the examination calendar of many medical and public health schools, while others were already on break. Second, our participation may have been limited as we only promoted the contest at one in-person AMR event. Third, although our contest was global in scope, our entries were not entirely representative of all global settings, as there were no entries from the Latin American region. Fourth, entries accepted through the online learning platforms were limited to those in the English language and Chinese (Ding Xiang Yuan). This may have affected participation from non-English and non-Chinese speaking countries. Also, due to the small sample size, future studies and data are needed to establish crowdsourcing as an approach to address medical education and AMR training.

## Conclusions

This study enhances our understanding of crowdsourcing in the context of medical education. Our contest demonstrates that crowdsourcing can be used to increase study materials available for medical students and physicians. Clinical educators could consider adopting crowdsourcing approaches to enhance medical education and mitigate traditional barriers associated with curriculum development. There is a need for additional research testing the impact and efficacy of crowdsourced clinical training resources for students and practitioners.

## Supplementary Information

**Additional file 1.** Additional figures and tables.

## Data Availability

Additional data collected from entries are available in additional files.
