# Supplementary material for: Crowdsourcing to develop open-access learning resources on antimicrobial resistance
Source: BMC Infect Dis. 2021 Sep 6;21:914. doi: 10.1186/s12879-021-06628-0 (PMC8419975; doi:10.1186/s12879-021-06628-0)
Supplement: Supplementary file 1 — Additional file 1. Additional figures and tables. [file 12879_2021_6628_MOESM1_ESM.docx]

**Crowdsourcing to develop open-access learning resources on antimicrobial resistance**

Eneyi E. Kpokiri*^1^, Randall John*^2^, Dan Wu^1^, Noah Fongwen^1^, Jehan Z. Budak^3^, Christina C. Chang^4^, Jason J. Ong^1,5^, Joseph D. Tucker^1,6,7^

*Contributed equally

^1^ Faculty of Infectious and Tropical Diseases, London School of Hygiene and Tropical Medicine, London, UK

^2^ Department of Health Policy and Management, Gillings School of Global Public Health, University of North Carolina, Chapel Hill, NC

^3^ Department of Medicine, Division of Allergy & Infectious Diseases, University of Washington, Seattle, WA

^4^ Partners ID Images, Department of Infectious Diseases, Massachusetts General Hospital, Boston, MA

^5^ Central Clinical School, Monash University, Australia

^6^ Social Entrepreneurship to Spur Health (SESH), Guangzhou, China

^7^ Institute of Global Health and Infectious Diseases, University of North Carolina, Chapel Hill, NC

Corresponding author:

Eneyi E. Kpokiri, Faculty of Infectious and Tropical Diseases, London School of Hygiene and Tropical Medicine, Keppel St., Bloomsbury, London, WC1E 7HT, United Kingdom

[eneyi.kpokiri@lshtm.ac.uk](about:blank)

**Additional Figure S1. Sample Promotional Infographic.**


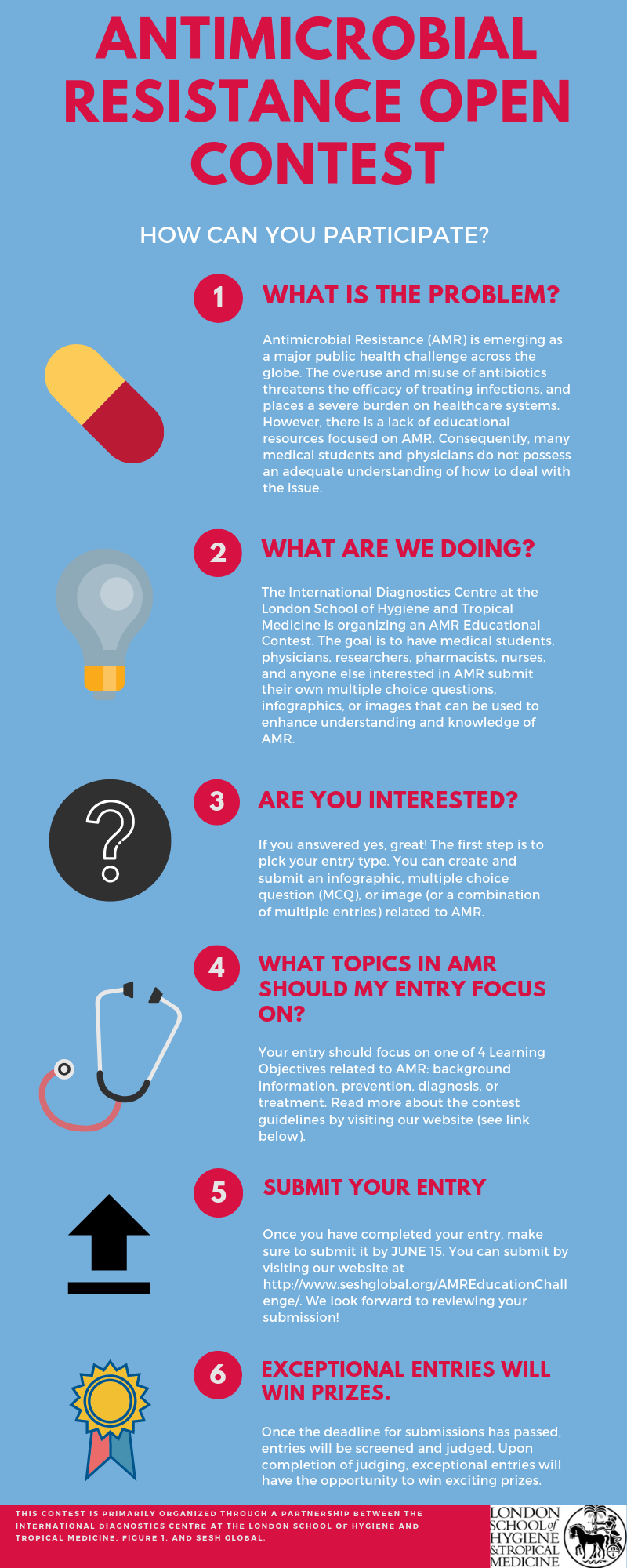


**Additional Figure S2. Finalist Infographic 1.**

**
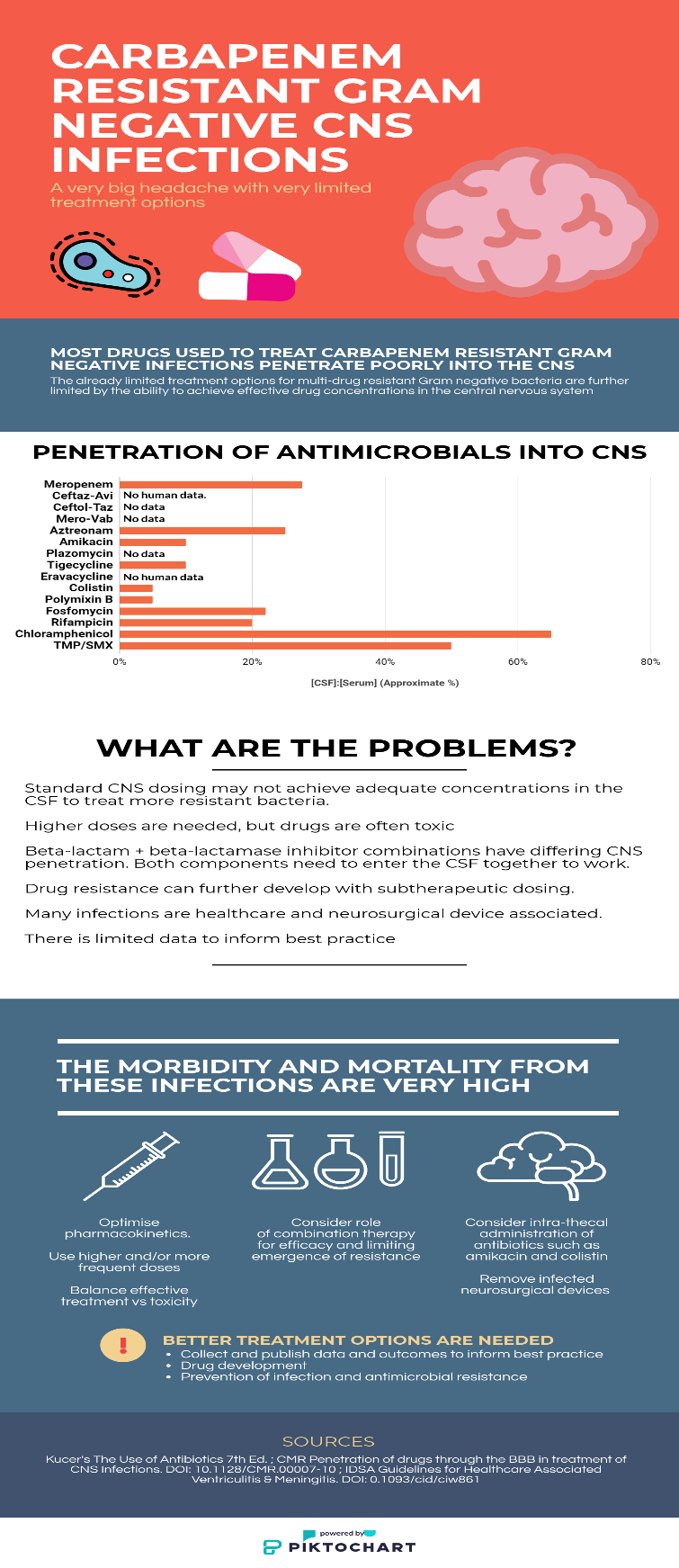
**

**Additional Figure S3. Finalist Infographic 2.**


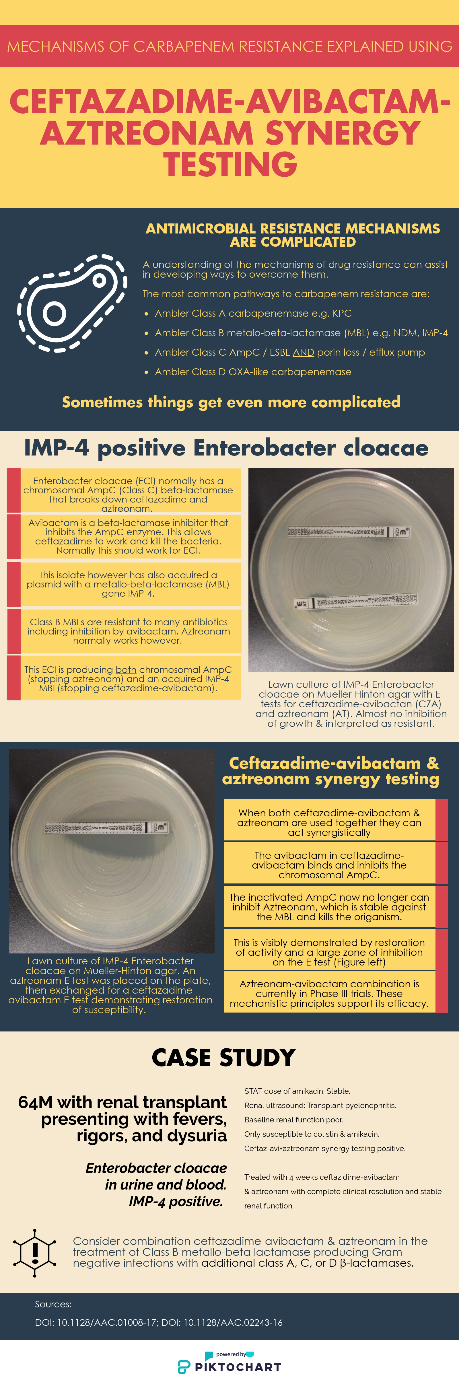


**Additional Figure S4. Finalist Infographic 3.**


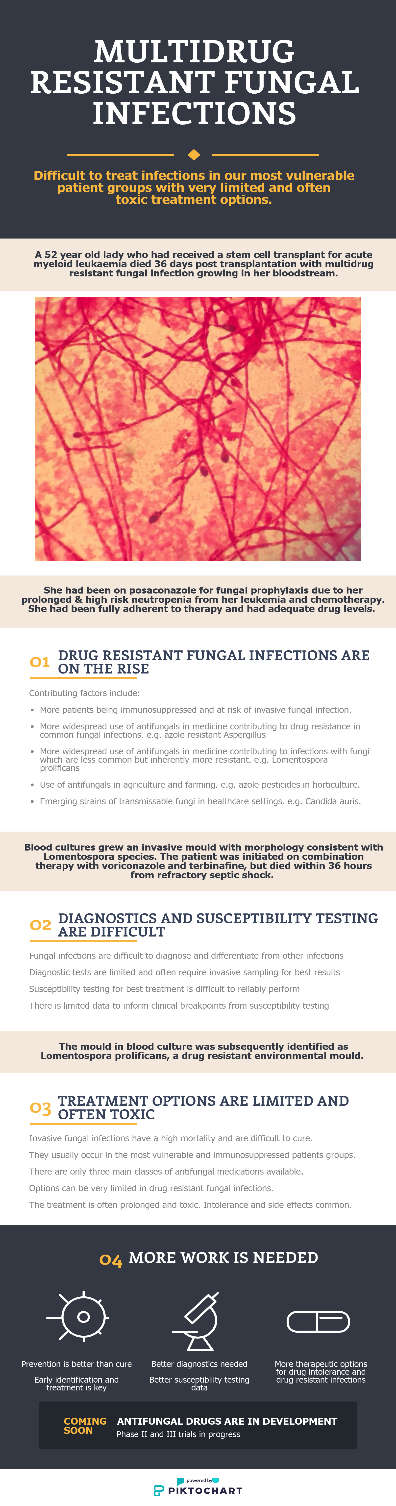


**Additional Table S1. Finalist Infographic Explanatory Captions.**

| **Infographic Number** | **Title** | **Explanatory Caption (Submitted by Finalists)** | **Country** |
| --- | --- | --- | --- |
| Finalist Infographic 1  (Additional Figure S2) | Carbapenem Resistant Gram-Negative CNS Infections | The submitted infographic highlights the great difficulties in management of central nervous system infections from carbapenem resistant gram-negative organisms. It displays simplified data on CNS penetration of commonly used antimicrobials in treatment of MDR Gram negatives. This serves to highlight both the paucity of data to inform practice and the limited options available. It subsequently discusses key problems in the area and offers some important concepts in managing these difficult infections. | Australia |
| Finalist Infographic 2 (Additional Figure S3) | Ceftazidime-Avibactam-Aztreonam Synergy Testing | The submitted infographic outlines the use of combination ceftazadime-avibactam and aztreonam in the treatment of metallobetalactamse producing carbapenem resistant gram-negative infections with additional production of a Class A/C/D beta lactamase. It serves a dual purpose as mechanistically explains the pathways of resistance and how they are inhibited (serving an educational role in teaching mechanisms of resistance) as well as highlight a potential therapeutic option in difficult to treat MDR infections, especially MBL producing infections. It additionally highlights an area of clinical use of aztreonam-avibactam currently in Phase III studies. | Australia |
| Finalist Infographic 3 (Additional Figure S4) | Multidrug Resistant Fungal Infections | The infographic shows an often-forgotten aspect of antimicrobial resistant - drug resistant fungal infections. In line with the ever-increasing problem of antibacterial resistance, the rates of antifungal drug resistance are rising. These are occurring in our most vulnerable patient groups and have high morbidity and mortality. Treatment options are limited and toxic. The infographic highlights both a simple case of drug resistant fungal infection, as well as highlights some key issues in managing these infections. | Australia |

**Additional Table S2. Finalist Multiple-Choice Questions (MCQs).**

| **MCQ Number** | **Question** | **Answer Choices** | **Correct Answer** | **Country** |
| --- | --- | --- | --- | --- |
| **Finalist MCQ 1** | To make infection control effective in a health institution, the following measures should be put in place except: | 1. Patients harboring resistant organisms should be isolated | B | Nigeria |
|  |  | 1. Frequent turnover of health staff |  |  |
|  |  | 1. Good hand hygiene practices |  |  |
|  |  | 1. Surveillance to identify carriers of resistant organisms |  |  |
| **Finalist MCQ 2** | ESKAPE organisms include: | 1. *Escherichia coli, Salmonella typhi, Klebsiella pneumonia, Acinetobacter baumannii, Proteus* *mirabilis, Enterobacter spp* | C | Nigeria |
|  |  | 1. *Enterococcus fecium, Shigella spp, Klebsiella pneumonia, Acinetobacter baumannii, Pseudomonas aeruginosa, Enterobacter spp* |  |  |
|  |  | 1. *Enterococcus fecium, Staphylococcus aureus, Klebsiella pneumonia, Acinetobacter baumannii, Pseudomonas aeruginosa, Enterobacter spp* |  |  |
|  |  | 1. *Enterococcus fecium, Staphylococcus aureus, Klebsiella pneumonia, Acinetobacter baumannii, Pseudomonas aeruginosa, Escherichia coli* |  |  |
| **Finalist MCQ 3** | The following problems can be linked to poor utilization of antibiotics in animal farming: | a. Residues in foodstuffs | D | Cameroon |
|  |  | b. Antimicrobial resistance |  |  |
|  |  | c. Drugs in the environment |  |  |
|  |  | d. All of the above |  |  |
|  |  |  |  |  |
| **Finalist MCQ 4** | If symptoms of an infection persist after initial treatment with antibiotics, what is the best option? | a. Increase the dose of the same antibiotic treatment | C | Cameroon |
|  |  | b. Prolong the duration of the same antibiotic treatment |  |  |
|  |  | c. Request a laboratory analysis (antibiogram)  d. Prescribe additional antibiotics |  |  |
|  |  | e. Change the antibiotic drug |  |  |
| **Finalist MCQ 5** | The following are mechanisms of antimicrobial resistance EXCEPT: | a. Enzymatic inactivation | D | Cameroon |
|  |  | b. Target modification |  |  |
|  |  | c. Efflux pumps |  |  |
|  |  | d. Programmed cell death |  |  |
|  |  | e. Metabolic alterations |  |  |
